# Profiling Adolescent Lifestyles and Their Sociodemographic Drivers: A School-Based Study from Rawalpindi, Pakistan

**DOI:** 10.3390/children12111507

**Published:** 2025-11-06

**Authors:** Humaira Mahmood, Babar Tasneem Shaikh, Azka Naseem, Farrah Pervaiz, Abdul Momin Rizwan Ahmad

**Affiliations:** 1 School of Public Health, Health Services Academy, Islamabad 44000, Pakistan; humairatalha@hotmail.com (H.M.); babar.shaikh@hsa.edu.pk (B.T.S.); dr.azkanaseem@gmail.com (A.N.); 2 Department of Community Medicine, Rawalpindi Medical University, Rawalpindi 44000, Pakistan; farrah.pervaiz@rmur.edu.pk; 3 Department of Human Nutrition and Dietetics, NUST School of Health Sciences, National University of Sciences & Technology (NUST), Sector H-12, Islamabad 44000, Pakistan; 4 Department of Health Sciences, University of York, York YO10 5DD, UK

**Keywords:** adolescent health, health-promoting behaviors, lifestyle behaviors, sociodemographic predictors, ALP-R2, Pakistan

## Abstract

**Highlights:**

**What are the main findings?**

- The lifestyle profiling of school-going adolescents showed three clearly defined clusters categorized as poor, moderate, and good in Rawalpindi, Pakistan.
- Among the seven explored domains, the highest scores were observed in positive life perspective and interpersonal relations, whereas nutrition, physical activity, and health responsibility were the lowest-scoring domains.

**What are the implications of the main finding?**

- To mitigate lifestyle-related risk factors, school-based interventions should prioritize gender and socioeconomic disparities.
- The findings show that an adolescent’s quality of life is influenced by multiple factors linked with their habits.

**Abstract:**

Background/Objectives: Adolescence is the phase of life when an individual develops habits that lead to their health outcomes in later life stages; yet, comprehensive evidence from low- and middle-income countries (LMICs) like Pakistan remains limited. This study examined the lifestyle behaviors of school-going adolescents in Rawalpindi and their correlation with key sociodemographic factors. Methods: A descriptive cross-sectional study was conducted in four (public and private) schools, using multistage cluster sampling (n = 675). Lifestyle behaviors were examined within seven predefined domains. K-means cluster analysis was used for the identification of distinct behavioral profiles. Associations with age, gender, and school type were examined using Chi-square tests. Correlations within clusters were analyzed using Pearson’s correlation test. Results: The majority of adolescents demonstrated positive life perspectives (88.3%) and strong interpersonal relationships (83.8%), while nutrition (16.2%), physical activity (31.7%), and health responsibility (15.0%) were weaker domains. Cluster analysis revealed three groups: poor (n = 129), moderate (n = 334), and good (n = 212) lifestyle behaviors. Statistically significant associations were found between lifestyle profiles and both age group (*p* = 0.037) and school type (*p* = 0.007), with private school students being more likely to exhibit healthier behaviors. Gender differences were notable in physical activity, but not significant in other domains. Conclusions: Interventions targeting low-performing domains, especially physical activity and nutrition, are needed—particularly for females and public school students. These findings highlight the importance of targeted, school-based lifestyle interventions in resource-limited settings.

## 1. Introduction

Adolescence, defined by the World Health Organization (WHO) as the period between 10 and 19 years of age, is a critical phase of human development that is marked by profound biological, psychological, and social transformations [1]. The behaviors and habits formed during this formative stage often persist into adulthood, profoundly influencing an individual’s long-term health trajectory. As adolescents begin to exercise greater autonomy over their lifestyle choices, such as diet, physical activity, sleep patterns, and social engagement, the need to understand and guide these behaviors becomes increasingly urgent [2].

Disease patterns have changed in recent decades, as the world continues to evolve. The social, economic, and environmental transitions, including improved sanitation, living standards, and advanced healthcare systems, are responsible for the shift from infectious, communicable diseases to non-communicable diseases (NCDs). It is observed that NCDs like cardiovascular diseases, diabetes, cancers, and chronic respiratory diseases have increased greatly [3,4]. As per the WHO report, 75% of global deaths are due to NCDs [5], with the majority occurring in low- and middle-income countries (LMICs), where healthcare systems are constrained by limited resources [6]. Despite the increase in life expectancy, most of the deaths reported occur prematurely, before the age of 70, due to the increasing prevalence of NCDs. Lifestyle-related risk factors, such as inadequate physical activity, poor dietary intake, substance abuse, and sedentary behaviors are found to be associated with these premature deaths [7]. Urbanization and technological advancement, a culture of convenience and sedentary living, is also becoming common, especially among the youth. Technological dependence has impacted adolescents’ lifestyles greatly. These poor lifestyle behaviors include poor dietary habits, reduced physical activity, prolonged screen time, and disturbed sleeping patterns, and have been found to be risk factors for the development of NCDs in later life [4,8]. The literature shows that most young individuals exhibit these poor lifestyle habits without being aware of the long-term effects on their overall health. Thus, early preventive measures, such as educational interventions and behavioral awareness programs are needed [9].

Like other lower-middle-income countries, Pakistan is also facing the challenges linked with a rapid epidemiological transition alongside economic instability. Unlike high-income countries, where challenges are different from the LMICs, their research data cannot be used for resolving such public health problems. A data set from 52 LMICs highlighted the underlying factors leading to the nutritional deficit amongst adolescents. The study indicated that consumption of convenience foods like junk food and carbonated drinks is higher, as it costs less than the healthier options [8]. In Pakistan, the prevalence of higher consumption of convenient food and lower intake of fruits and vegetables among the adolescents is nearly 21%, with almost 31% exposed to more than two suboptimal dietary factors [8]. Another systematic review reported that Pakistan is experiencing the double burden of malnutrition among school-going adolescents, as these dietary patterns do not meet the recommended intake [10]. The latest national survey reports have triggered warnings about the substantially increasing prevalence of hypertension and diabetes in the population, which are due to unhealthy behaviors adopted in the early phases of life [11]. Factors such as limited health literacy, socioeconomic disparities, urbanization, and inadequate public health infrastructures further exacerbate the issue. Despite this, research exploring comprehensive lifestyle behaviors among Pakistani adolescents remains limited, especially in studies examining socio-demographic variations [12].

Most of the research studies conducted in LMICs have primarily considered individual behaviors, like physical activity or dietary intake alone, leaving other significantly influencing behaviors undiscovered. Owing to different natures of the challenges existing in LMICs, especially Pakistan, the study results conducted in high-income countries are not applicable. For developing a contextually appropriate approach for handling the prevailing problems, it is important to build a comprehensive understanding of how an individual’s lifestyle is influenced by multiple factors, including physical, social, and spiritual aspects, along with sociodemographic variance in the population [13,14,15]. To conceptually frame these relationships, this study draws on the social ecological model (SEM), which emphasizes that health behaviors are shaped by interrelated factors, involving multiple levels—individual, interpersonal, organizational, community, and policy [16]. This framework highlights how adolescent lifestyle practices are influenced beyond individual behaviors. Building on the work of Harper et al. [17], the SEM further emphasizes the need to identify barriers and facilitators to adolescent health across interconnected levels, from individual awareness to enabling policy environments.

This study encompasses the existing literature gaps by examining the lifestyle profiles of school-going adolescents in Rawalpindi, Pakistan, along with their associations with socio-demographic factors. The study has highlighted modifiable behaviors and sociodemographic disparities, leading to evidence for developing more culturally acceptable public health strategies that will help to enhance the quality of life for adolescents and reducing the burden of NCDs on the healthcare system through cost-effective approaches. The study outcomes have also provided insightful findings that can engage all stakeholders, including policymakers, educators, healthcare providers, and parents/guardians, who play roles in achieving the targets that are aligned with the sustainable developmental goals (SDG 3.4) that are related to NCD-associated premature mortality rates and are controlled through preventive measures. This study provides a framework for efficient, cost-effective and sustainable interventions that can help Pakistan to improve the health of young generations.

## 2. Materials and Methods

### 2.1. Study Design

A descriptive, cross-sectional study conducted in schools.

### 2.2. Study Settings

The research was conducted in Rawalpindi, an urban district in northern Punjab, Pakistan. Rawalpindi offers a diverse socioeconomic and educational landscape, characterized by a mix of public and private educational institutions that reflect both urban and peri-urban living conditions. Public schools in this region primarily cater to middle- and low-income families, often facing infrastructural limitations, while private institutions serve higher-income populations and tend to have better academic and extra-curricular resources. This heterogeneous context makes Rawalpindi an ideal site for studying adolescent health behaviors in relation to varying socioeconomic determinants.

### 2.3. Study Population and Sampling Strategy

The study’s targeted population consisted of school-going adolescents studying in grades 6 to 10 in public and private schools in Rawalpindi, Pakistan. A multistage sampling strategy was employed for selecting schools. All the schools that fulfilled the inclusion criteria (pupil strength more than 500, co-educational, conducting secondary school grades from 6 to 10) were identified. Initially, 15 eligible schools were contacted, out of which 8 were willing to participate, and of these, 4 (2 each from public and private) were selected through purposive sampling, considering the maximum variation to ensure demographic variation. For student enrollment in each school, cluster sampling was used, whereby all the students present on the data collection day were invited to participate. The final sample size was calculated as 675, accounting for a 19% prevalence of adolescent overweight/obesity [18], 95% confidence level, 4% margin of error, design effect of 1.5, and a 20% non-response rate.

### 2.4. Data Collection Tool

Lifestyle behaviors were assessed using the Adolescent Lifestyle Profile-Revised 2 (ALP-R2) questionnaire, a validated instrument originally developed by Hendricks, Murdaugh, and Pender to measure health-promoting behaviors in adolescents [19]. The tool includes demographic information and evaluates seven domains of adolescent health-promoting behaviors: nutrition, physical activity, health responsibility, stress management, interpersonal relationships, spiritual growth, and positive life perspective. Each domain is assessed through a set of Likert-scale items (ranging from “never” to “always”), with higher scores indicating greater engagement in health-promoting behaviors. The tool is self-administered and structured, for the ease of completion by adolescents.

The psychometric soundness of ALP-R2 has been confirmed in various international settings, including Chile (n = 572) [20], Turkey (n = 890) [21], and Colombia (n = 1476) [22]. Previous studies demonstrated high internal consistency (e.g., Ω = 0.87) and construct validity across diverse adolescent populations [20,23]. For the present study, the suitability of the ALP-R2 tool for the Pakistani population was confirmed by exploratory factor analysis (EFA) and a reliability test. Factor extraction was guided by eigenvalues and scree plots; the adequacy of the sample was determined by the Kaiser–Meyer–Olkin (KMO) measure and Bartlett’s test of sphericity. Internal consistency was checked by using Cronbach’s alpha (α = 0.890), which exceeds the acceptable threshold of ≥0.70.

### 2.5. Data Collection Procedure

Before proceeding with the data collection procedure, permission from the administration of the selected schools was obtained through the official channel. After the approval, teachers and the other school staff were given a demonstration explaining the purpose and procedure of the study. The consent of parents was obtained prior to the commencemnt of the study. While ensuring minimal disruption to the routine activities of the school, the data were collected within regular school hours. On the scheduled day of data collection, trained data collectors visited the school, gave a brief demonstration of how to complete the questionnaire, and the questionnaire was administered, after obtaining consent for participation from the students. The data collectors were available at the site throughout to provide any assistance required while filling out the questionnaires and ensured proper completeion of the forms.

After the participants completed the forms, they were collected and reviewed for completeness, and securely dispatched to the research office for data entry and analysis. Anonymity and confidentiality of the recorded information was fully ensured throughout the process.

### 2.6. Data Analysis

All data were analyzed using SPSS version 26. Descriptive statistics (means, standard deviations, frequencies) summarized participants’ lifestyle behaviors and demographic profiles. To identify the distinct patterns of lifestyle behaviors among adolescents, a K-means cluster analysis was conducted, using standardized scores from the seven ALP-R2 subscales. Following cluster formation, Pearson’s correlation analysis was performed within each cluster to examine the interrelationships among the ALP-R2 subscales. Additionally, Chi-square tests of independence were used to examine the associations between cluster membership and key sociodemographic variables, including age group, gender, and school type. A significance level of *p* < 0.05 was applied to all inferential tests.

## 3. Results

### 3.1. Descriptives

The study included a total of 675 adolescents, with a mean age of 14.8 years (SD = 1.7). Of the total sample, 43% were early adolescents (10–14 years) and 57% were late adolescents (15–18 years). The gender distribution was relatively balanced, with 51.3% male and 48.7% female participants. In terms of school type, 56.9% attended private schools, while 43.1% were enrolled in government schools. Participants were drawn from grades 6 to 10.

To better understand adolescents’ lifestyle behaviors, responses from the ALP-R2 instrument were initially collected on a 5-point Likert scale (1 = never, 5 = almost always). For each participant, the mean scores were computed across the seven subscales and summed to obtain a total lifestyle score. The overall mean ALP-R2 score was 24.13 (SD = 3.74). These total scores were used to categorize participants into three behavior levels: poor (1.00–2.49), moderate (2.50–3.49), and good (3.50–5.00) [24].

Table 1 presents the mean and standard deviation for each ALP-R2 subscale across age groups, gender, and school type. Across all groups, positive life perspective consistently showed the highest mean scores, indicating a strong sense of optimism among participants. Physical activity scores were higher among males (M = 3.54) and government school students (M = 3.46), whereas females (M = 2.95) scored lower in this area. Nutrition and health responsibility demonstrated relatively lower scores across most groups, while spiritual health was the highest among private school students (M = 3.87). Stress management scores were moderate and relatively consistent across demographic groups.

Table 2 summarizes the distribution of participants across behavior levels for each subscale. The majority reported good behaviors in positive life perspective (88.3%), interpersonal relationships (83.8%), and spiritual health (59.9%). However, fewer adolescents reported good behaviors in nutrition (16.2%), health responsibility (15.0%), and physical activity (31.7%), with a notable 30.2% being categorized as poor in physical activity. Stress management was mostly moderate, with 54.2% falling into the moderate category and 34.1% into the good category.

### 3.2. Cluster Analysis

A K-means cluster analysis was conducted using standardized scores from the seven ALP-R2 subscales to identify distinct patterns in adolescent lifestyle behaviors. The algorithm converged after 10 iterations, producing a three-cluster solution:

- Cluster 1 (n = 129): Poor lifestyle behaviors, scoring low across all domains.
- Cluster 2 (n = 334): Moderate lifestyle behaviors, scoring near average.
- Cluster 3 (n = 212): Good lifestyle behaviors, scoring high in all domains.

Table 3 displays the standardized mean scores (Z-scores) across the subscales for each cluster. These clusters represent meaningful groupings of adolescents with similar lifestyle profiles, supporting the application of the ALP-R2 tool in this context.

### 3.3. Intra-Cluster Analysis

To explore the internal relationships among the lifestyle domains, Pearson’s correlation analyses were conducted separately within each cluster.

- Cluster 1 (poor behavior): Moderate positive correlations were found between nutrition and health responsibility (r = 0.31, *p* < 0.001), and between positive life perspective and both physical activity (r = 0.29, *p* = 0.001) and spiritual health (r = 0.23, *p* = 0.010). Other correlations were weak or nonsignificant.
- Cluster 2 (moderate behavior): Several weak but significant negative correlations emerged. For example, nutrition was negatively correlated with physical activity (r = −0.18, *p* = 0.001), interpersonal relations (r = −0.020, *p* < 0.001), and stress management (r = −0.22, *p* < 0.001).
- Cluster 3 (good behavior): Positive associations were observed among several domains. For instance, positive life perspective was significantly correlated with interpersonal relations (r = 0.27, *p* < 0.001) and stress management (r = 0.20, *p* = 0.004). A weak negative correlation was found between physical activity and interpersonal relations (r = −0.16, *p* = 0.023).

### 3.4. Inferential Analysis

**Figure 1:**
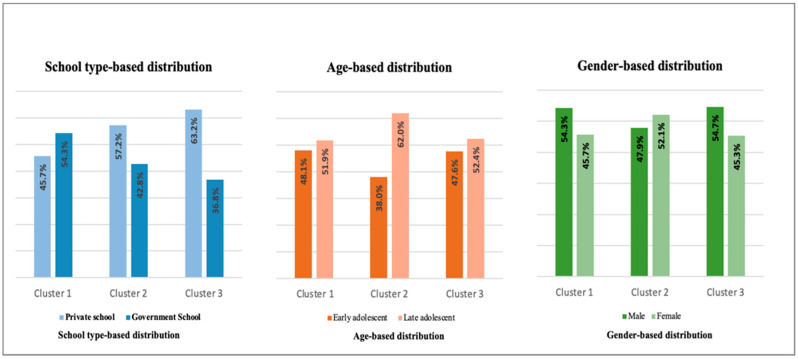
Distribution of lifestyle behavior clusters across demographics.

Chi-square tests of independence were conducted to examine the association between demographic characteristics and lifestyle behavior clusters. As presented in Table 4 and Figure 1, a statistically significant association was observed between age group (*p* = 0.037) and school type (*p* = 0.007) with cluster membership. The distribution indicated that a larger proportion of late adolescents tended to fall into the moderate behavior cluster, while private school students were more likely to belong to the good behavior cluster. In contrast, adolescents attending government schools were predominantly represented in the poor behavior cluster. Meanwhile, the gender-based distribution appeared relatively even across clusters, with no statistically significant association observed (*p* = 0.225).

### 3.5. Regression Analysis

Multiple linear regressions were performed to assess whether age group, gender, and school type predicted each of the seven ALP-R2 lifestyle themes. No significant associations were found for nutrition and stress management.

Significant models emerged for positive life perspective, health responsibility, physical activity, spiritual health, and interpersonal relationships. Among these, school type was a consistent predictor, particularly for spiritual health (β = 0.323) and positive life perspective (β = 0.141). Gender significantly predicted physical activity (β = −0.260) and health responsibility (β = 0.118), while age group was inversely associated with health responsibility (β = −0.097). Complete regression coefficients and model statistics are presented in Table 5.

## 4. Discussion

The study findings showed that most of the adolescents in our study population had strongly positive life perspectives and interpersonal relationships. However, they exhibited a significantly lower nutritional profile, and lower physical activity and factors linked with health responsibility. Three clearly outlined clusters were identified and categorized as poor, moderate, and good. The gender-wise profile difference was evident in the physical activity domain, where male members were observed to have significantly higher scores than females. Considering the types of schools, students from the private sector were more likely to have a healthier profile, whereas a large proportion of students from the public sector fell into the poor profile cluster. The study results highlight the socioeconomic and demographic variations linked with lifestyle profiles in Pakistan.

Among the seven domains assessed, the lowest scores were observed in nutrition, physical activity, and health responsibility. These findings are supported by the evidence from other studies, which reported the higher prevalence of poor dietary habits and sedentary lifestyles among adolescents worldwide [20,25]. The current research findings on nutritional profiles align with a cross-sectional study conducted on high school pupils in Saudia Arabia, where more than half of the participants had a poor diet quality and a higher consumption of processed foods, including snacks and drinks [26]. It is equally concerning that physical activity scores were low across the demographic groups, particularly among females and private school students. This is part of a widespread trend—over 81% of adolescents globally fail to meet the WHO’s recommendation of at least 60 min of moderate-to-vigorous activity daily [27]. In Pakistan, nearly 59% of the youth lead sedentary lifestyles, regularly exceeding 4 h of screen time [28]. This aligns with the current study findings that physical activity was one of the poorest performing domains.

Within Pakistan, similar trends have been reported in other provinces. For instance, a study conducted in Khyber Pakhtunkhwa indicated lower physical activity amongst urban adolescents with no gender difference when compared to adolescents living in rural areas [29]. Another cross-sectional study in the Punjab province showed that physical activity among school-going adolescents was linked to the physical education activities conducted in schools. It emphasized that provision of physical education within schools can help to reduce obesity in school-going adolescents [12].

The gender variable stood as a significant predictor in this study for the physical activity domain, where males scored higher, and in health responsibility, in which girls were more responsible. The gender-based difference in physical activity observed in this study is consistent with the outcomes of multiple large-scale studies [30,31]. Research consistently shows that male adolescents are more likely to meet physical activity guidelines, with higher engagement in moderate-to-vigorous physical activity (MVPA) [32]. For instance, a study assessing adolescents in China showed that MVPA in boys was markedly higher than in girls [33]. Another study examining the young population of India also reported that girls residing in urban cities are more likely to adapt a sedentary lifestyle [34]. These findings across the Asian population are accounted for by the social norms, safety concerns, and community mindset, wherein girls are encouraged to participate in households more than engaging in outdoor co-curricular activities. These results highlight the necessity to develop more contextually appropriate strategies, catering to gender disparities that are prevalent in such societies.

The existing literature persistently supports a positive correlation between socioeconomic status (SES) and the adoption of health-promoting behaviors in adolescents. Higher SES gives better accessibility and affordability for resources like healthcare services, healthy food, and higher educational attainment. Therefore, those with higher SES tend to exhibit better health outcomes and an improved quality of life [35,36,37,38]. With respect to the present study’s findings, school type acts as proxy indicator of SES. In the educational system of Pakistan, public schools run by the government often represent the students from lower-income families, as fee structures are affordable for this class of the society. On the other hand, the private sector, based on high-quality education, comes at a higher cost, attracting those belonging to higher socioeconomic backgrounds. This status difference is well represented in the findings, where good profile clusters were composed largely of those studying in private schools, while poor clusters were disproportionately composed of public school students. These results suggest that socioeconomic disparities may play a significant role in shaping adolescent lifestyle behaviors.

Cluster patterns defining the distinct behaviors of adolescents have been reported in previous studies, which, to clarify the present study’s cluster analysis, explain how an individual is influenced by multifactorial aspects. For example, a large-scale study of Ireland also reported the presence of multiple clusters based on behavioral patterns reflecting the demographic variance among study participants. The researchers of the study, based on their findings, encouraged the development of targeted interventions that could help to reduce health-related disparities and improve behavioral deficits [39].

In this paper, the outcomes of the regression analysis showed that domains such as positive life perspective, spiritual health, and interpersonal relationships were significantly affected by the school type. This is also supported by the findings of another study in Chile, which assessed the adolescent profile using the same tool (ALP-R2), where those adolescents with high SES exhibited better health profiles in all domains [20].

This study provides strong support for a broader consideration of multiple lifestyle-related factors. SEM, based on multifaceted interactions at various levels (individual, interpersonal, organizational, and the community), serves a good framework for designing strategies that account for all the demographic groups, including gender, age, and school type, and for facilitating effective school-based health promotion programs.

The major limitation of our study is its cross-sectional design, which hinders the establishment of causal relation between the lifestyle profile and sociodemographic characteristics. The presence of recall bias and social desirability also affects the data collected, which is based on a self-administered questionnaire. Also, the study only examined the profiles of adolescents enrolled in schools; those outside the schools were not considered, which affects the generalizability of the findings. In view of the above-discussed limitations, it is recommended that longitudinal studies should be considered for future research, so as to cater for long-term changes in behaviors and in-depth assessments of psychosocial factors. In addition to the study design, incorporation of intervention based on the present findings can also help with gathering data on tailored interventional programs for mitigating the prevailing poor lifestyle behaviors among the targeted population.

## 5. Conclusions

The lifestyle profile of school-going adolescents in Rawalpindi, Pakistan, was examined using ALP-R2. The findings of the paper provided a thorough overview of various aspects of an adolescent’s lifestyle and its association with various sociodemographic variables. Among the seven domains, positive life perspective and interpersonal relationships demonstrated overall good scores, whereas nutrition, physical activity, and health responsibility persistently scored less. Among the clusters identified as poor, moderate, and good, participants from public schools exhibited lower scoring, largely falling into the poor cluster. Physical activity was also observed to be disproportionately distributed among the genders, with boys scoring higher than girls. Despite the study’s limitations, the outcomes contribute significantly to the local context, indicating the need to conduct further research into this domain with more robust designs.

## Figures and Tables

**Table 1:** Mean and standard deviation of ALP-R2 subscale scores by demographics (N = 675).

Themes	Age				Gender				School Type			
	Early Adolescent N = 290		Late Adolescent N = 385		Male N = 346		Female N = 329		Government N = 291		Private N = 384	
	Mean	SD	Mean	SD	Mean	SD	Mean	SD	Mean	SD	Mean	SD
Nutrition	3.04	0.79	3.01	0.73	2.99	0.76	3.05	0.76	2.97	0.76	3.06	0.76
Physical Activity	3.24	1.18	3.27	1.03	3.54	1.01	2.95	1.09	3.46	1.06	3.09	1.09
Health Responsibility	3.07	0.81	2.89	0.71	2.87	0.80	3.07	0.70	2.97	0.79	2.96	0.74
Positive Life Perspective	4.04	0.78	4.00	0.61	4.02	0.69	4.02	0.67	3.91	0.73	4.10	0.64
Interpersonal Relations	3.93	0.71	3.86	0.68	3.82	0.76	3.96	0.61	3.79	0.72	3.97	0.66
Stress Management	3.29	0.89	3.42	0.73	3.41	0.81	3.31	0.79	3.34	0.73	3.38	0.86
Spiritual Health	3.67	0.93	3.59	0.89	3.63	0.94	3.62	0.88	3.30	0.88	3.87	0.86

**Table 2:** Descriptives of adolescent lifestyle profile, theme-wise (N = 675).

Theme	Poor (n, %)	Moderate (n, %)	Good (n, %)
Nutrition	79 (23.7%)	201 (60.2%)	54 (16.2%)
Physical Activity	101 (30.2%)	127 (38.0%)	106 (31.7%)
Health Responsibility	91 (27.2%)	193 (57.8%)	50 (15.0%)
Positive Life Perspective	1 (0.3%)	38 (11.4%)	295 (88.3%)
Interpersonal Relation	3 (0.9%)	51 (15.3%)	280 (83.8%)
Stress Management	39 (11.7%)	181 (54.2%)	114 (34.1%)
Spiritual Health	22 (6.6%)	112 (33.5%)	200 (59.9%)

**Table 3:** K-means cluster (Z scores) analysis.

Variable	Cluster 1	Cluster 2	Cluster 3
Total (N) (675)	129	334	212
Nutrition	−0.83	−0.17	0.77
Physical Activity	−0.55	−0.25	0.72
Health Responsibility	−0.95	−0.13	0.79
Positive Life Perspective	−1.30	0.11	0.61
Interpersonal Relations	−1.24	0.02	0.71
Stress Management	−0.92	−0.12	0.75
Spiritual Health	−0.91	−0.06	0.66

**Table 4:** Chi-square results for each demographic variable.

Demographic Variable	χ^2^ Value	Df	*p*-Value
Age Group (Early/Late)	6.59	2	0.037 *
Gender (Male/Female)	2.99	2	0.225
School Type (Govt/Private)	10.00	2	0.007 *

* *p*-Value ≤ 0.05 is statistically significant.

**Table 5:** Theme-wise regression analysis summary.

Theme	R^2^	F (df)	*p* (Model)	β (Age)	*p*	β (Gender)	*p*	β (School Type)	*p*
Nutrition	0.005	1.05 (3, 671)	0.370	−0.007	0.862	0.029	0.470	0.055	0.165
Physical Activity	0.091	22.37 (3, 671)	0.000 *	−0.062	0.104	−0.260	0.000 *	−0.128	0.001 *
Health Responsibility	0.027	6.29 (3, 671)	0.000 *	−0.097	0.013 *	0.118	0.003 *	−0.0043	0.0271
Positive Perspective	0.020	4.49 (3, 671)	0.004 *	−0.010	0.804	−0.024	0.547	0.141	0.000 *
Interpersonal Relations	0.023	5.20 (3, 671)	0.001 *	−0.016	0.687	0.077	0.054	0.111	0.004 *
Stress Management	0.010	2.27 (3, 671)	0.080	0.071	0.074	−0.057	0.156	0.041	0.295
Spiritual Health	0.102	25.54 (3, 671)	0.000 *	−0.018	0.624	−0.068	0.074	0.323	0.000 *

* *p*-Value ≤ 0.05 is statistically significant.

## Data Availability

The data presented in this study are available on request from the corresponding author due to the institutional’s policies.

## Abbreviations

The following abbreviations are used in this manuscript:

ALP-R2	Adolescent Lifestyle Profile-Revised 2
EFA	Exploratory Factor Analysis
WHO	World Health Organization
NCDs	Non-Communicable Diseases
LMICs	Low- and Middle-Income Countries
KMO	Kaiser–Meyer–Olkin
SDG	Sustainable Development Goal
CFA	Confirmatory Factor Analysis
MVPA	Moderate-to-Vigorous Physical Activity
SES	Socioeconomic Status
SEM	Social Ecological Model
